# Pharmacological Modulation of the Psychiatric Risk Factor FKBP51 Alters Efficiency of Common Antidepressant Drugs

**DOI:** 10.3389/fnbeh.2018.00262

**Published:** 2018-11-12

**Authors:** Max L. Pöhlmann, Alexander S. Häusl, Daniela Harbich, Georgia Balsevich, Clara Engelhardt, Xixi Feng, Michaela Breitsamer, Felix Hausch, Gerhard Winter, Mathias V. Schmidt

**Affiliations:** ^1^Department Stress Neurobiology and Neurogenetics, Max Planck Institute of Psychiatry, Munich, Germany; ^2^Department of Pharmacy, Pharmaceutical Technology and Biopharmaceutics, Ludwig-Maximilians-Universität, Munich, Germany; ^3^Department of Chemistry, Technical University Darmstadt, Darmstadt, Germany

**Keywords:** FKBP51, fkbp5, anxiety, depression, stress, antidepressant, co-medication

## Abstract

Despite a growing body of research over the last few decades, mental disorders, including anxiety disorders or depression, are still one of the most prevalent and hardest to treat health burdens worldwide. Since pharmacological treatment with a single drug is often rather ineffective, approaches such as co-medication with functionally diverse antidepressants (ADs) have been discussed and tried more recently. Besides classical ADs, there is a growing number of candidate targets identified as potential starting points for new treatment methods. One of these candidates, the FK506 binding protein 51 (FKBP51) is linked to a number of psychiatric disorders in humans. In this study, we used SAFit2—a newly developed modulator of FKBP51, which has shown promising results in rodent models for stress-related disorders delivered in a depot formulation. We combined SAFit2 with the commonly prescribed selective serotonin reuptake inhibitor (SSRI) escitalopram and performed basic behavioral characterization in a mouse model. Remarkably, co-application of SAFit2 lowered the efficacy of escitalopram in anxiety-related tests but improved stress coping behavior. Given the fact that mental diseases such as anxiety disorders or depression can be divided into different sub-categories, some of which more or less prone to stress, SAFit2 could indeed be a highly beneficial co-medication in very specific cases. This study could be a first, promising step towards the use of FKBP51 modulators as potent and specific enhancers of AD efficiency for subclasses of patients in the future.

## Introduction

In recent years, our understanding and perception of mental disorders, like anxiety disorders or depression, as a major burden for our society, has increased tremendously. It is well known that some of these illnesses are highly prevalent and account for immense costs to health care systems all over the world (Ferrari et al., 2013). To make matters worse, the efficiency of available treatments, especially pharmacological interventions, is often very low and individual response to a specific treatment regimen is unpredictable (Hirschfeld, 1999). Additionally, most of the available pharmacological treatments are based on the same principle, namely modulating the levels of neurotransmitters in the brain.

The majority of the widely prescribed antidepressants (ADs), like paroxetine or escitalopram, alter the availability of monoamines like serotonin in the synaptic cleft. Although these so-called selective serotonin reuptake inhibitors (SSRIs) prove to be effective in patients with anxiety disorders as well as major depression, the effect size is modest and there is a high non-responder rate (Jakobsen et al., 2017). The late onset and high relapse rates of these drugs are additive drawbacks, making therapy a tedious trial and error process (Rush et al., 2006). The lack of a mechanistically broader drug spectrum makes it hard to treat non-responsive patients, and so-called “treatment-resistant” patients often lack a real chance of recovery. For this reason, focus has shifted towards a broad cluster of cellular mechanisms and pathways linked to mental disorders as well as approaches that combine different methods and treatments to achieve more reliable results. General anxiety disorder, for example, is often treated with common SSRIs alone; however, AD response can be enhanced by psychotherapy (Strawn et al., 2018).

One of the main pathways of interest in relation to major depression is the hypothalamic-pituitary-adrenal (HPA) axis, which is a key player in many stress-related disorders. Its regulation is known to be crucial for mental health (de Kloet et al., 2005) and many promising targets for the treatment of mental diseases are mediators of HPA axis regulation (Ben-Efraim et al., 2011; Kang et al., 2018; Stonawski et al., 2018). Two of the key regulatory components of the HPA axis are the high affinity mineralocorticoid receptor (MR) and the lower affinity glucocorticoid receptor (GR), which mediate the glucocorticoid negative feedback of the HPA axis and therefore an appropriate termination of the stress response (Ulrich-Lai and Herman, 2009). Altered glucocorticoid signaling and thus impaired HPA-axis activity has been observed in many patients with mood and anxiety disorders (Pariante and Miller, 2001). Both MR and GR are present in the cell as part of a larger protein complex, which includes HSP90 and a number of regulatory proteins. One of these proteins is the co-chaperone FK506 binding protein 51 (FKBP51), which reduces binding sensitivity of the GR to its ligand (Pratt et al., 2006; Criado-Marrero et al., 2018). Interestingly, polymorphisms in the regulatory regions of the gene encoding FKBP51 (called fkbp5) are associated with depression and post-traumatic stress disorder in humans (Binder et al., 2004, 2008).

FKBP51 has become one of the top candidates for novel interventions and treatments for stress-related disorders and as a result there is a rapidly growing body of preclinical data on FKBP51. Indeed, Scharf et al. (2011) showed that FKBP51 is regulated in a stress-dependent manner in several brain regions, including the hippocampus, the paraventricular nucleus (PVN) of the hypothalamus and the amygdala. Conventional FKBP51 knockout (KO) mice are less affected by chronic social defeat stress (Hartmann et al., 2012) and FKBP51 signaling in the amygdala is crucially involved in anxiety-related behavior in mice (Hartmann et al., 2015). SAFit2, the first potent, brain permeable and highly specific inhibitor of FKBP51 was recently developed and was shown to increase neuroendocrine feedback and reduce anxiety in mice (Gaali et al., 2015).

For further development of FKBP51 antagonists as potential ADs and anxiolytics, it is important to understand whether pharmacological FKBP51 inhibition interferes with the action of commonly prescribed ADs, which are also often prescribed for anxiety disorders. Although combining ADs increases the risk of an uncontrolled aggravation of negative side effects, co-medication can improve symptoms in some patients (Thase, 2006). As FKBP51 KO mice are less responsive to the acute effects of an SSRI treatment (Gassen et al., 2014), it is important to assess whether the pharmacological inhibition of FKBP51 might alter the efficacy of SSRI treatment. Therefore, in this study, we investigated the effects of escitalopram with or without FKBP51 inhibition on anxiety and stress coping behavior.

## Materials and Methods

### Animals

Animals used for this experiment were 12-week-old male mice, bred on a C57BL/6N background in our in-house facilities. Testing was carried out in the animal facilities of the Max-Planck-Institute of Psychiatry (Munich, Germany). To ensure sufficient acclimation, the animals were single housed and moved from our holding rooms to the test room 2 weeks prior to the experiment. All animals were housed in standard cages (21 cm × 15 cm × 14 cm, Plexiglas) and conditions were kept constant during the whole experiment (light-dark-cycle 12 h:12 h, lights on at 08:00 am, 23 ± 2°C, humidity 55%). The animals had *ad libitum* access to food (Altromin 1324, Altromin GmbH, Germany) and tap water.

The experiment was carried out in accordance with the European Communities Council Directive 2010/63/EU. All animal suffering was minimized during the testing. The protocols were approved by the committee for the Care and Use of Laboratory Animals of the Government of Upper Bavaria, Germany.

### Experimental Design

**Figure 1:**
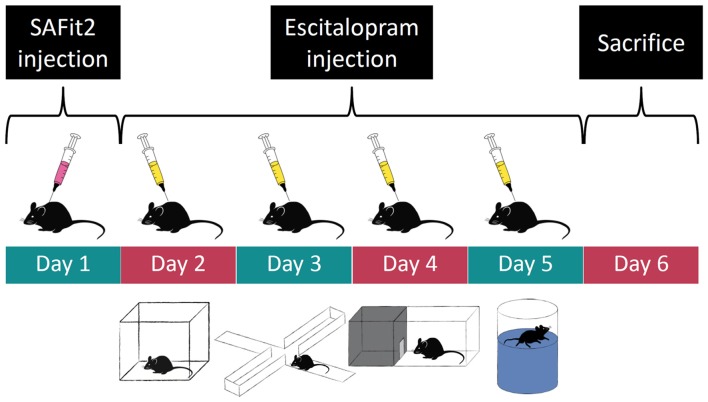
Experimental time course: order of pharmacological treatment and behavioral tests. Empty and SAFit2 loaded vesicular phospholipid gel (VPG) was injected subcutaneously on day 1. Behavioral tests were carried out on days 2–5 with a single intraperitoneal vehicle or escitalopram injection 30 min prior to each test.

We used a 2 × 2 design with four experimental groups: (1) control animals, injected with empty vesicular phospholipid gel (VPG) and vehicle; (2) SAFit2 animals injected with SAFit2 VPG and vehicle; (3) escitalopram animals, injected with empty VPG and escitalopram; and (4) SAFit2+escitalopram animals, receiving both drugs. The initial group size was *n* = 10 for each group. On day 1 of the experiment (see a graphical representation of the experimental design in Figure 1), half of the animals received SAFit2 treatment, and the other half received an injection of the empty VPG (see details for “Pharmacological Treatment” section below). On the subsequent days, half of each group received either the SSRI escitalopram or vehicle 30 min before behavioral testing. The following tests were performed, always with a 24-h inter-trial interval: Open Field (OF), Elevated Plus Maze (EPM), Dark-Light Box (DaLi) and Forced Swim Test (FST). The animals were sacrificed 24 h after the last behavioral test.

### Pharmacological Treatment

SAFit2 was applied using a VPG as a carrier as described previously (Balsevich et al., 2017; Maiarù et al., 2018) where it was shown to result in stable plasma and brain SAFit2 concentrations with a drug half-life of about 7 days. Briefly, 200 μl of the VPG with or without SAFit2 (gel loading at 10 mg SAFit2 per g gel) were injected subcutaneously on day 1 of the experiment. Escitalopram was obtained from Santa Cruz Biotechnology as a crystalline powder and dissolved in H_2_O to a final concentration of 1 mg/ml. The drug was injected intraperitoneally 30 min prior to testing on experimental days 2–5. The administered dose was 10 mg/kg body weight and the injection volume was corrected to the animal’s bodyweight accordingly.

## Behavioral Tests

All behavioral tests were carried out between 08:30 am and 12:30 pm in the same room the animals were housed in. All tests were analyzed by an experienced researcher utilizing the automated video-tracking software AnyMaze (Anymaze 4.99, Stoelting, Wood Dale, IL, USA). All animals underwent the same testing battery in the same order of tests. To minimize possible carryover effects of the different behavioral tests, the order of tests was arranged from the least stressful to the most stressful (McIlwain et al., 2001).

### Open Field Test (OF)

The OF was performed on day 2 in order to measure locomotion as well as anxiety behavior in the animals. Testing was conducted under low light conditions (~15 lux) in an empty arena (50 cm × 50 cm × 50 cm) made of gray polyvinyl chloride (PVC). At the beginning of the test, animals were placed in one of the corners of the apparatus. The total test time was 15 min, divided into three time bins of 5 min each. For this test, parameters of interest were the distance traveled, time spent immobile, as well as the time animals spent investigating the most exposed and therefore aversive mid-section (10 cm × 10 cm) of the arena (Hartmann et al., 2015).

### Elevated Plus Maze (EPM)

The EPM was performed on day 3 of the experiment. The maze consisted of two opposed open arms (30 cm × 5 cm × 0.5 cm) and two opposed enclosed arms (30 cm × 5 cm × 15 cm) made of gray PVC, which were connected by a central platform (5 cm × 5 cm) shaping a plus sign. The arena was elevated 50 cm above the floor. At the start of the test, animals were placed into the center of the plus maze facing a closed arm and were allowed to explore the maze for 5 min. The time spent in the respective arms as well as the number of open arm entries was analyzed. Animals that fell off the open arm of the apparatus during testing were excluded from the analysis (Müller et al., 2003).

### Dark-Light Box (DaLi)

The DaLi was performed on day 4 of the experiment and served as another paradigm to measure anxiety-related behavior. The arena consisted of a rectangular box that was split into two compartments. The compartments were connected by a 4 cm long tunnel, allowing the animals to move freely between both compartments. The lit compartment (30 cm × 20 cm × 25 cm) was illuminated by an external light source (~700 lux), creating an aversive environment for the animals. The dark compartment (15 cm × 20 cm × 25 cm) on the other hand was not illuminated (~5 lux). The time the animals spent in each chamber during the 5 min test duration was measured (Hartmann et al., 2015).

### Forced Swim Test (FST)

The FST was conducted on the last day of the experiment, as it is considered to be the most stressful of all the tests. For the FST, we used 2-liter glass beakers that were filled up to 1.5 liter with water at room temperature. Water depth was chosen in a way that the mice were neither able to touch the bottom of the container, nor climb out of it, therefore creating an inevitable stressful situation for the animals. Test duration was 5 min and the animals were dried with a towel afterwards to prevent hypothermia. The scored parameter was the time spent in active escape behavior (struggling; Gaali et al., 2015).

### Corticosterone

Blood samples were taken by tail cut 30 min (stress response) and 90 min (stress recovery) after the onset of the FST. Basal blood samples were taken from trunk blood at sacrifice 24 h after the FST. All samples were collected in 1.5 ml EDTA-coated microcentrifuge tubes (Kabe Labortechnik, Germany). Blood samples were kept on ice and later centrifuged at 8,000 rpm at 4°C for 15 min. Plasma was transferred to new, labeled tubes and stored at −20°C until determination of corticosterone by radioimmunoassay (MP Biomedicals Inc.; sensitivity 12.5 ng/ml).

### Statistical Analysis

The data are shown as means ± SEM, analyzed by the commercially available software GraphPad Prism 7.03. Students’ *t*-test was employed for comparison of two independent groups. Two-factorial (FKBP51 antagonist and AD) ANOVA was employed for all other parameters. A significance level of *p* < 0.05 for main effects and *p* < 0.1 for interaction effects was followed by Tukey’s *post hoc* test, with a nominal level of *p* < 0.05 considered significant. All values outside a margin of two times standard deviation were considered outliers and excluded from the analysis. Investigators were blinded to the experimental groups during the experiments and data analysis.

## Results

### Open Field

**Figure 2:**
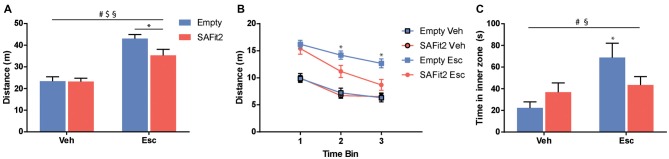
Behavioral alterations in the open field (OF) test. **(A)** Escitalopram (Esc) administration induced an increase in distance traveled during the test, which was significantly ameliorated by SAFit2 treatment. **(B)** When splitting the test in three time-bins of 5 min each, we found no differences between the Esc groups in the first 5 min. However, in time bins 2 and 3, co-medication significantly decreased the distance traveled when compared to the Empty/Esc group. **(C)** The exploration of the inner zone of the OF is considered a measure of anxiety. Esc significantly increased the inner zone time of the animals, and this effect was blocked in SAFit2 treated mice. ^#^Main Esc effect, ^$^main SAFit2 effect, ^§^Esc–SAFit2 interaction effect, *significant *post hoc* effect at *p* < 0.05.

The OF is classically used to examine locomotor activity and anxiety-like behavior. For the total distance traveled (Figure 2A), ANOVA revealed no main effect of SAFit2 treatment (*F*_(1,36)_ = 3.567, *p* < 0.1), but a main effect of escitalopram (*F*_(1,36)_ = 56.03, *p* < 0.001) as well as an interaction effect (*F*_(1,36)_ = 3.196, *p* < 0.1). Under vehicle treated conditions, SAFit2 had no effect on overall locomotion. Escitalopram treatment significantly increased locomotor activity in both groups. However, the escitalopram effect was significantly reduced in SAFit2 treated animals compared to empty VPG treated controls (*p* < 0.05). When the data were split up in three 5-min time bins (Figure 2B), it becomes clear that the moderating effect of SAFit2 treatment on the escitalopram effect is mainly evident in the 2nd (*p* < 0.05) and 3rd (*p* < 0.05) time bin.

Regarding the time the animals spent in the inner zone of the OF (Figure 2C), ANOVA revealed no main effect of SAFit2 (*F*_(1,34)_ = 0.3393, *p* > 0.05), but a main effect of escitalopram (*F*_(1,34)_ = 7.862, *p* < 0.01) as well as a significant interaction effect (*F*_(1,34)_ = 4.402, *p* < 0.05). In animals without SAFit2, escitalopram application resulted in a marked anxiolytic effect, indicated by the increased time spent in the inner zone of the OF (*p* < 0.05). This effect was absent in animals treated with SAFit2.

### Elevated Plus Maze

**Figure 3:**
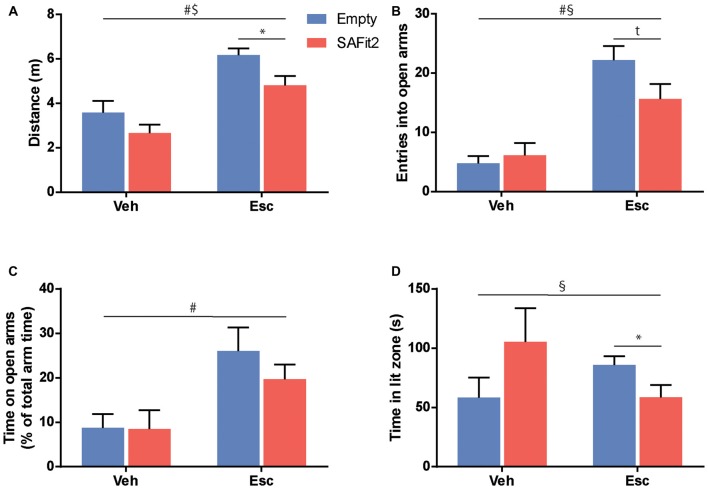
Treatment with SAFit2 and escitalopram (Esc) affected behavior in two different anxiety tests. **(A)** Overall locomotion in the Elevated Plus Maze (EPM) was increased by Esc treatment and this effect was ameliorated by SAFit2. Anxiety-like behavior in the EPM measured by open arm entries **(B)** and open arm time **(C)** was decreased after Esc treatment. When combined with the FK506 binding protein 51 (FKBP51) inhibitor, this effect was reduced for open arm entries, but not significantly for open arm time. **(D)** In the Dark-Light box (DaLi), we again observed a significant interaction effect when comparing all four groups. ^#^Main Esc effect, ^$^main SAFit2 effect, ^§^Esc–SAFit2 interaction effect, *significant *post hoc* effect at *p* < 0.05, ^t^*post hoc* trend at *p* < 0.1.

To test for anxiety-related and explorative behavior we employed the EPM. Here, ANOVA revealed that SAFit2 (*F*_(1,33)_ = 7.26, *p* < 0.05) and escitalopram (*F*_(1,33)_ = 31.41, *p* < 0.001) treatment resulted in significant main effects in distance traveled. However, there was no interaction between the four treatment groups (*F*_(1,33)_ = 0.2775, *p* > 0.1). In vehicle-treated animals, SAFit2 had no significant effect on locomotion (*p* > 0.05), but in escitalopram treated mice SAFit2 significantly reduced locomotor activity compared to empty gel treated mice (*p* < 0.05; Figure 3A).

SAFit2 did not induce a significant main effect when looking at anxiety-related parameters e.g., the number of entries into the open arm (*F*_(1,33)_ = 1.521, *p* > 0.05; Figure 3B) or the time animals spent on the open arms (*F*_(1,33)_ = 0.658, *p* > 0.05; Figure 3C). As expected, animals treated with escitalopram had a significant increase in open arm entries (*F*_(1,33)_ = 39.45, *p* < 0.001) and time spent (*F*_(1,33)_ = 12.39, *p* < 0.001). Again, this anxiolytic effect was moderately dampened when escitalopram was combined with SAFit2, as depicted by a trend towards a lower number of entries into the open arms (*p* < 0.1; Figure 3B).

### Dark-Light Box

Neither SAFit2, nor escitalopram induced significant main effects on explorative behavior in the DaLi (escitalopram: *F*_(1,33)_ = 0.1, *p* > 0.05; SAFit2: *F*_(1,33)_ = 0.038, *p* > 0.05). Nonetheless, we saw a significant interaction effect, using Two-Way ANOVA (*F*_(1,33)_ = 5.838, *p* < 0.05; Figure 3D). In line with our former observations, SAFit2 co-medication significantly reduced the anxiolytic effect of escitalopram treatment (*p* < 0.05).

### Forced Swim Test

**Figure 4:**
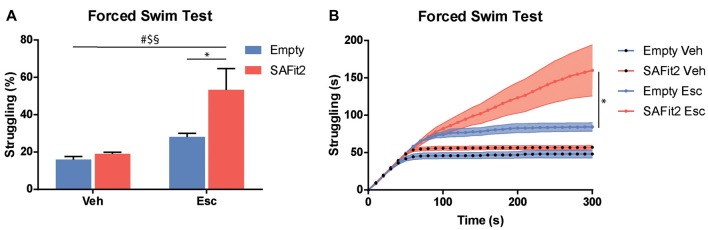
SAFit2 and escitalopram (Esc) treatment strongly affect behavior in the Forced Swim Test (FST). **(A)** Percentage of time the animals spent struggling in the FST was significantly increased by both SAFit2, as well as Esc treatment. When both drugs were combined, animals spent more than 50% of the time actively trying to escape the water. **(B)** When looking at the time course of the 5-min experiment, it becomes apparent that animals with a SAFit2 pre-treatment struggled significantly more in the last 3 min of the FST. ^#^Main Esc effect, ^$^main SAFit2 effect, ^§^Esc–SAFit2 interaction effect, *significant *post hoc* effect at *p* < 0.05.

The FST is a recognized test to evaluate stress coping behavior in rodents, as it confronts them with an unescapable stressful situation. ANOVA revealed significant main effects for SAFit2 (*F*_(1, 32)_ = 7.21, *p* < 0.05) and escitalopram (*F*_(1, 32)_ = 19.55, *p* < 0.001), as well as an interaction effect (*F*_(1, 32)_ = 4.474, *p* < 0.05), when looking at the time animals spent struggling (Figures 4A,B). SAFit2 alone had no effect on struggling behavior. As expected, escitalopram treatment significantly increased time spent struggling in both groups. Interestingly, SAFit2 treatment significantly enhanced this effect when considering the full testing time (Figure 4A, *p* < 0.05). When analyzing the progression of the struggling behavior over time, repeated measures ANOVA revealed a significant time * escitalopram * SAFit2 effect (*F*_(3,33)_ = 2.947, *p* < 0.001). *Post hoc* analysis showed that SAFit2 pretreatment significantly enhanced escitalopram-induced struggling behavior especially in the last 4 min of the FST (*p* < 0.01).

### Corticosterone

**Figure 5:**
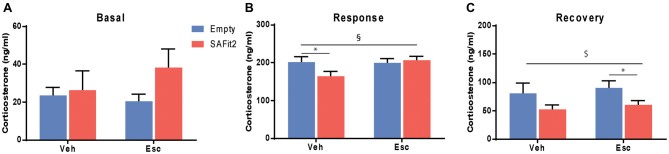
Effects on hypothalamic-pituitary-adrenal (HPA) axis function. **(A)** Basal corticosterone secretion is not affected by SAFit2 or escitalopram (Esc) treatment. **(B)** Thirty minutes following an acute stressor (response), SAFit2 reduces corticosterone secretion in vehicle treated animals, while no effect is observed under Esc treatment. **(C)** Ninety minutes after the onset of the stressor (recovery), SAFit2 suppression of the HPA axis function is observed in both vehicle and Esc-treated animals, but only reaches *post hoc* significance in the Esc-treated group. ^$^Main SAFit2 effect, ^§^Esc–SAFit2 interaction effect, *significant *post hoc* effect at *p* < 0.05.

Corticosterone was measured at baseline and following an acute stressor (forced swim) to determine HPA axis responsivity and feedback. Under basal conditions, there were no main (escitalopram: *F*_(1,33)_ = 0.33, *p* > 0.05, SAFit2: *F*_(1,33)_ = 1.82, *p* > 0.05) or interaction (*F*_(1,33)_ = 0.98, *p* > 0.05) effects (Figure 5A). Following an acute stressor, ANOVA revealed no main effects (escitalopram: *F*_(1,33)_ = 2.434, *p* > 0.05, SAFit2: *F*_(1,33)_ = 1.389, *p* > 0.05), but a significant interaction effect (*F*_(1,33)_ = 3.239, *p* < 0.1; Figure 5B). SAFit2 pretreatment significantly reduced the corticosterone response in vehicle treated animals (*p* < 0.05), but not in escitalopram treated animals (*p* > 0.05). At 90 min after the onset of the stressor (recovery), we observed a significant SAFit2 effect (*F*_(1,33)_ = 5.481, *p* < 0.05), but no escitalopram (*F*_(1,33)_ = 0.458, *p* > 0.05) or interaction (*F*_(1,33)_ = 0.002, *p* > 0.05) effect. SAFit2 pretreatment lowered the corticosterone recovery levels, an effect that was stronger in escitalopram-treated animals (*p* < 0.05), while it did not reach *post hoc* significance in vehicle treated animals (*p* > 0.05; Figure 5C).

## Discussion

In terms of pharmacological intervention, SSRIs are a physician’s main weapon against mental illnesses like anxiety disorders or major depressive disorder. However, their efficacy is mediocre at best and we are in urgent need of alternatives. In this study, we further investigated the potential use of the FKBP51 inhibiting substance SAFit2, which targets a different molecular mechanism. While SSRIs directly impact the amount of available neurotransmitters in the synaptic cleft, SAFit2 regulates the termination of the body’s stress response, which is often dysfunctional across mental illnesses. In addition, FKBP51 has been shown to regulate several protein-protein interactions important for cellular function, which may be affected by SAFit2 treatment (Gassen et al., 2015; Balsevich et al., 2017). Here, we combined these two treatment approaches in hope of obtaining a broader spectrum of beneficial properties that may help more patients.

Our results suggest a very relevant interaction between escitalopram and SAFit2. When applied exclusively, SAFit2 had very little impact on behavior. This might be due to the relatively low dose used via the continuous release VPG formulation. In addition, all tests were performed under basal conditions. From earlier experiments we know that SAFit2 induces (positive) behavioral effects on anxiety-related behavior when used at higher doses or when combined with a stressful challenge (Hartmann et al., 2015). In contrast, escitalopram treatment resulted in robust behavioral alterations in the animals, increasing their overall mobility and readiness to assume risk and enhancing active stress coping behaviors. For anxiety-related behavior, the addition of SAFit2 dampened the anxiolytic effects of escitalopram, without canceling them out completely. While an increase in neurotransmitter levels via SSRI action reduces anxiety and fear, simultaneous disruption of FKBP51 functionality seems to counteract this effect. This finding is in line with the clinical observation that FKBP51 risk allele carriers with high FKBP51 levels show an improved response to SSRI treatment (Ellsworth et al., 2013). In addition, FKBP51 KO mice were previously shown to respond less to SSRI treatment (Gassen et al., 2014). In contrast to our observations in the anxiety-related parameters, the addition of SAFit2 enhanced the effects of AD application in the FST. In agreement with former studies (Can et al., 2012), active stress coping in the FST was significantly enhanced after escitalopram treatment, with the effect being even larger in the presence of SAFit2.

The converse results in our experiments clearly demonstrate how delicate and specific the outcome of co-medication can be. In the current study a combination of serotonin reuptake inhibition with FKBP51 blockade might be beneficial for the treatment of symptoms that relate to stress coping, while positive AD effects on anxiety-related parameters can be hampered by the disruption of FKBP51 functionality. One might speculate that the given challenge in each test is mediated by different pathways or systems in the brain, which are ultimately differentially affected by our combination of drugs. As we confirmed the previously reported suppression of HPA axis reactivity following SAFit2 treatment, it is possible that the behavioral effects of SSRI treatment are modulated via the altered HPA axis function. However, basal corticosterone levels were not different between the groups. Furthermore, SSRIs have been shown to affect a plethora of cellular pathways, some of which seem independent of their function as SSRI (Einoch et al., 2017; Eskelund et al., 2017). Similarly, the molecular functions of FKBP51 also largely exceed its role as Hsp90/GR co-chaperone (Balsevich et al., 2017; Fries et al., 2017; Hamilton et al., 2018). Indeed, SAFit2 likely affects only some of the actions of FKBP51, while others may not be modified. Thus, it is likely that with the development of additional FKBP51-modulating drugs different aspects of FKBP51 function can be targeted, which may enhance the applicability of co-medication with SSRIs.

The current study also has a number of limitations. Importantly the effects of SSRI treatment were only tested following an acute treatment, while some SSRI-mediated effects e.g., on anxiety-related behavior occur predominantly following chronic treatment (Burghardt and Bauer, 2013). In addition, only one class of ADs was tested. In the future, it will therefore be important to test more combinations of various ADs with SAFit2 following both acute and chronic treatment regiments. Given the fact that we found positive effects on active stress coping behavior in the FST, switching to an AD that affects the dopamine or norepinephrine system could be a promising course of action in future experiments (Bardal et al., 2011).

Overall, our results show the potential promise but also the potential problems and limitations of co-medication when it comes to the treatment of mental disorders. They also highlight the importance and necessity of pre-clinical studies to determine whether or not a chosen combination of drugs has the potential to help patients. In conclusion, this study provides an important characterization of the therapeutic capabilities of the newly developed drug SAFit2 and its potential impact on classical pharmacological treatments in the future.

## Author Contributions

MP and MS designed the experiments and wrote the manuscript. MP, AH, DH, GB, CE and MS performed the experiments. XF and FH synthesized the FKBP51 antagonist. MB and GW developed the VPG and incorporated the FKBP51 antagonist in the VPG. MP analyzed the data.

## Conflict of Interest Statement

The authors declare that the research was conducted in the absence of any commercial or financial relationships that could be construed as a potential conflict of interest.
